# Egg consumption reduces the risk of depressive symptoms in the elderly: findings from a 6-year cohort study

**DOI:** 10.1186/s12888-023-04540-2

**Published:** 2023-01-17

**Authors:** Fudong Li, Xiaoli Li, Xue Gu, Tao Zhang, Le Xu, Junfen Lin, Kun Chen

**Affiliations:** 1 Department of Epidemiology and Biostatistics, School of Medicine, Zhejiang University, 866 Yuhangtang Road, Xihu District, Hangzhou, 310058 China; 2 Zhejiang Provincial Center for Disease Control and Prevention, Zhejiang, Hangzhou China; 3 The Second Affiliated Hospital of Zhejiang University School of Medicine, Zhejiang, Hangzhou China

**Keywords:** Depressive symptoms, Egg consumption, Cohort study, Elderly

## Abstract

**Background:**

Accumulating evidence showed that dietary habits might modify the risk of depression. This study aimed to evaluate the longitudinal association of egg consumption with depressive symptoms in the Chinese elderly.

**Methods:**

We analyzed the data from Zhejiang Ageing and Health Cohort Study including 8289 participants. The Patient Health Questionnaire-9 scale (PHQ-9) was used to assess depressive symptoms at baseline and three waves of follow-up (2015, 2016, and 2019–2020). A PHQ-9 cut-off score ≥ 5 was used to define depressive symptoms. The participants with depressive symptoms at baseline were excluded. Egg consumption was evaluated through the diet habits section of the baseline questionnaire. Self-reported egg consumption was measured as the number of eggs per week and categorized into three categories. Log-binomial regression models with Generalized Estimating Equations were utilized to evaluate the association of egg consumption with depressive symptoms and estimate relative risks (RRs).

**Results:**

The mean age of included participants was 68.6 years. After 6 years of follow-up, 1385 (16.7%) participants were indicated with depressive symptoms by PHQ-9 at least once. Compared with non-consumers or less-than-weekly consumers, participants consuming < 3 eggs/week and ≥ 3 eggs/week had 30% (RR = 0.70, 95%CI 0.62–0.80) and 38% (RR = 0.62, 95%CI 0.54–0.71) lower risks of depressive symptoms, respectively. A linear association was confirmed (P for trend < 0.01), and each egg increment per week was associated with a 4% lower risk of depressive symptoms (RR = 0.96, 95%CI 0.93–0.99). Sensitivity analyses yielded consistent results to the main analyses.

**Conclusions:**

Egg consumption is prospectively related to a lower risk of depressive symptoms in the Chinese elderly. More prospective studies are needed to verify the association.

**Supplementary Information:**

The online version contains supplementary material available at 10.1186/s12888-023-04540-2.

## Background

Depression is one of the most common mental disorders around the world [1]. Based on estimation, 5% of adults suffer from depressive disorder worldwide [2], and it will be ranked third among disorders contributing to the global burden of disease by 2030 [3]. Moreover, depression is suggested the most frequent cause of emotional suffering in later life and significantly decreases the quality of life in older adults [4].

Accumulating evidence showed that dietary changes may favor the improvement or worsening of depression [5]. Eggs are a prominent source of high-quality protein, many vitamins, and bioactive components such as omega-3 fatty acids [6]. Those nutrients have been reported to benefit mood and mental well-being [7–9]. Animal studies demonstrated that orally administered eggs presented antidepressant-like effects [10]. However, relevant epidemiological studies remained scarce and inconsistent. One cross-sectional study reported that eating eggs more than once a week might have a beneficial effect on depressive symptoms [11]. On the contrary, another study demonstrated no significant association between egg intake and depressive symptoms [12]. Additionally, omega-3 egg intake was found associated with a lower likelihood of depressive symptoms in the subgroup of males only [13]. The lack of data from prospective studies makes it difficult to determine the causal relationship. Moreover, it needs to be further explored whether the association can be modified by other factors. It is noteworthy that no relevant study has been conducted in the Chinese population, while the culinary versatility of eggs [14] across different cultures (western vs. eastern) may also lead to differences in the association between egg consumption and depressive symptoms.

In this study, we utilized the data from a large population-based cohort study to investigate the longitudinal association of egg consumption with the risk of depressive symptoms among the Chinese elderly.

## Methods

### Data source

The data was from Zhejiang Ageing and Health Cohort Study, a community-based cohort study focusing on aging and health among the elderly in Zhejiang province, China [15]. It was conducted by Zhejiang Provincial Center for Disease Control and Prevention. Seven counties were randomly selected from a total of 90 counties in Zhejiang province. One town in each county and several communities in each town were then randomly selected. All permanent residents aged ≥60 years old in these selected communities were eligible and expected to be included in the study. In total, 13,955 individuals were eligible in the selected communities. The baseline survey was initiated in 2014, and 10,901 elderly were recruited and completed the survey (10,911 were primarily documented in the database, but 10 records were found duplicated later and were ultimately deleted). Three waves of follow-up surveys have been administered in 2015, 2016, and 2019–2020, especially. Due to funding restraint, the second wave of follow-up survey was administered in six counties, while other waves of follow-up surveys were administered in all seven counties.

A face-to-face interview based on a self-designed questionnaire was performed by trained research assistants for each participant at the baseline survey and each wave of the follow-up survey. The questionnaire included information on demographic characteristics, family status, reproductive history, medical disease, behavioral habits, diet habits, injury, depressive symptoms, self-care ability, and cognitive function. Data were checked by staff at Zhejiang Provincial Center for Disease Control and Prevention. Missing data and logical errors were fed back to the initial interviewers who would try to complete the dataset by reinvestigating the participants.

### Selection of participants

Ten thousand nine hundred one participants were enrolled in the baseline survey of the Zhejiang Ageing and Health Cohort Study. Participants with depressive symptoms at baseline (*n* = 1107) as well as those with incomplete baseline items in the questions related to depressive symptoms (*n* = 7) and egg consumption (*n* = 4) were excluded from the dataset. Moreover, participants with the presence of cognitive impairment (*n* = 1173) at baseline were excluded due to the higher possibility of recall bias. It was examined through the mini-mental state examination (MMSE) in the cognitive function section within the questionnaire, with a lower score indicating a poorer cognitive function. The widely accepted cut-off point to define cognitive impairment in China (MMSE Chinese Standard) is education-specific [16]: 17/18 for people with lower than primary education level, 20/21 for people with primary education level, 24/25 for people with higher than primary education level. Finally, a cohort for the association between egg consumption and risk of depressive symptoms included 8610 participants.

**Fig. 1:**
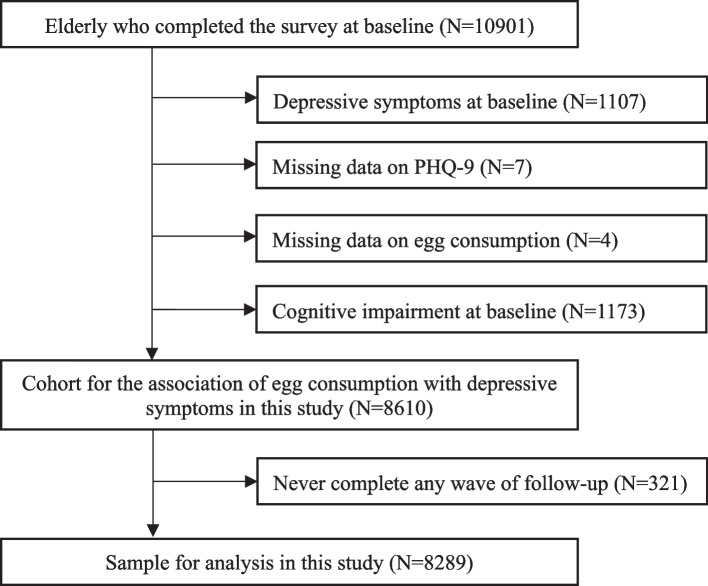
Flow diagram of the sample for analysis of the association between egg consumption and risk of depressive symptoms

Among them, 7575 (88.0%) completed the first wave of the follow-up survey. In the second wave of follow-up, 5791 (79.7%) from six counties completed the survey. 7203 (83.6%) completed the third wave of follow-up. Overall, more than half (4806, 55.8%) completed all three waves of the follow-up, 2668 (31.0%) completed two waves of the follow-up, 815 (9.5%) completed only one wave of the follow-up, and 321 (3.7%) never completed any wave of the follow-up. Hence, we included 8289 (95.8%) who attended the follow-up survey at least once in the final analysis (Fig. 1).

### Assessment of depressive symptoms

Patient Health Questionnaire-9 scale (PHQ-9), a 9-question version of the Primary Care Evaluation of Mental Disorders, was adopted to evaluate depressive symptoms [17]. The total score for the nine items ranges from 0 to 27, with greater values indicating increased severity. Scores of 5, 10, 15, and 20 represented cut-off points for mild, moderate, moderately severe, and severe depressive symptoms, respectively. The PHQ-9 has been validated with acceptable psychometric properties for depression screening in the Chinese population [18]. Our purpose was to identify participants with mild to severe depressive symptoms in the elderly. Therefore, a cut-off score of five or more was used to define depressive symptoms in this study.

### Egg consumption

Egg consumption at baseline was regarded as an exposure variable. Two items in the diet habits section of the baseline questionnaire were related to egg consumption: frequency of egg intake (days/week, “how many days did you have eggs every week generally in the last 12 months?”) and quantity of egg intake (eggs/day, “how many eggs did you have in the days you consumed eggs?”) [15].

Egg consumption (eggs/week) was calculated from frequency of egg intake multiplied by quantity of egg intake. We categorized participants into three groups according to their egg consumption: none or not weekly, < 3 eggs/week, and ≥ 3 eggs/week.

### Covariates

Based on findings reported in the literature, variables from baseline survey described below were considered as potential confounders in our analysis: age (years, continuous variable), gender (“male” and “female”), race (“Han ethnicity” and “minority”), education level (“lower than primary”, “primary”, “junior middle”, “senior middle” and “college and above”), marital status (“single”, “married” and “divorced/widowed”), family income (“≤10,000”, “10,001 ~ 20,000”, “20,001 ~ 50,000”, “50,001 ~ 100,000” and “> 100,000” Chinese Yuan/year), body mass index (“< 18.5”, “18.5 ~ < 24” and “≥24” kg/m^2^), hypertension (“presence” and “absence”), diabetes (“presence” and “absence”), coronary heart disease (“presence” and “absence”), smoking (“never”, “past” and “current”), alcohol drinking (“never”, “past” and “current”), exercise (“yes” and “no”), tea drinking (“yes” and “no”), vegetable intake (“< 7” and “≥7” days/week), fruit intake (“< 3” and “≥3” days/week), red meat intake (“< 3” and “≥3” days/week), fish intake (“< 3” and “≥3” days/week). Detailed information on some of these variables was described as follows.

(1) Medical disease section of the questionnaire contained the items on the presence or absence of 16 common diseases, which are supposed to be formally diagnosed by a physician. Hypertension, diabetes, and coronary heart disease (CHD) were considered in this study. (2) The frequency of other food intake was also collected in the diet habits section of the questionnaire. Vegetables, fruits, red meat, and fish were included in this study. It was divided into two categories for each variable based on the median. (3) Han ethnicity is the major ethnicity in the region.

### Statistical analysis

Descriptive statistics were applied to illustrate the general characteristics of included participants. The associations between general characteristics and egg consumption were examined by ANOVA, Kruskal-Wallis test, or Chi-square test as appropriate to variables. We used the log-binomial regression models for repeated measures with the Generalized Estimating Equations (GEE) method [19] to assess the longitudinal effect of egg consumption on the risk of depressive symptoms. Crude relative risks (RRs), 95% confidence intervals (CIs), and corresponding *P* values of depressive symptoms associated with egg consumption were calculated in model 1. In model 2, we adjusted for sociodemographic characteristics including age, gender, race, education level, marital status, and family income. In model 3, we additionally adjusted for BMI, hypertension, diabetes, coronary heart disease, smoking, alcohol drinking, exercise, and tea drinking. In model 4, vegetable intake, fruit intake, red meat intake, and fish intake were additionally adjusted. When the log-binomial model failed to converge, COPY method was adopted using a newly expanded data set that contained c-1 copies (c is usually set as 1000) of the original data and 1 copy of the original data with the dependent variable values interchanged [20]. Tests for linear trends were simultaneously implemented by assigning the median values to each category of egg consumption and modeling these values as a single continuous variable. Once the linear trend has been detected, the RR for each egg increment per week was calculated with the continuous variable of egg consumption (before transformation into the categorical variable) in the model. Furthermore, we also conducted stratified analyses to test whether observed associations varied by frequency of other food intakes. Meanwhile, interactions between egg consumption and these factors were checked through the addition of cross-product terms in the corresponding main-effects models. Finally, sensitivity analyses were adopted to evaluate the robustness of our results by 1) restricting the participants to those completing at least two waves of follow-up survey; 2) including the participants with depressive symptoms at baseline in GEE models, whose dependent variable value is expected to be similar (classified as depressive symptoms) at each wave of follow-up; 3) using the PHQ-9 score ≥ 9 to define depressive symptoms, which is generally accepted to screen for depression in China.

Statistical analyses were conducted using SAS Software Version 9.4 (SAS Institute Inc., Cary, NC, USA). A *P* value < 0.05 was considered statistically significant.

## Results

In the sample of 8289 participants (excluding depressive symptoms at baseline), 1385 (16.7%) participants were defined with depressive symptoms by PHQ-9 at least once during 6 years of follow-up. Participants’ baseline characteristics stratified by incident depressive symptoms are presented in Table 1. The mean age of participants was 68.6 years, with a lower mean age in those without depressive symptoms (*P* < 0.01). The risk of depressive symptoms was significantly associated with general characteristics including gender, race, marital status, and family income. Additionally, differences in hypertension, diabetes, CHD, smoking, alcohol drinking, exercise, and diet intake (vegetables, fruits, red meat, and fish) across categories of incident depressive symptoms were statistically significant. Nevertheless, the risk of depressive symptoms was not associated with education level (*P* = 0.12), BMI (*P* = 0.17), and tea drinking (*P* = 0.07). Table S1 shows participants’ baseline characteristics according to different categories of egg consumption.

**Table 1:** Baseline characteristics of 8289 included participants according to incident depressive symptoms

Variables	Total (***N*** = 8289)	Depressive symptoms		***P*** value
		No (***N*** = 6904)	Yes (***N*** = 1385)	
**Age** (years, Mean ± SD)	68.6 ± 7.0	68.3 ± 6.8	69.7 ± 7.3	< 0.01
**Gender** (N, %)				< 0.01
Male	4174 (50.4)	3549 (51.4)	625 (45.1)	
Female	4115 (49.6)	3355 (48.6)	760 (54.9)	
**Race** (N, %)				< 0.01
Han ethnicity	8060 (97.2)	6732 (97.5)	1328 (95.9)	
Minority	229 (2.8)	172 (2.5)	57 (4.1)	
**Education level** (N, %)				0.12
Lower than primary	3792 (45.8)	3122 (45.2)	670 (48.4)	
Primary	3714 (44.8)	3113 (45.1)	601 (43.4)	
Junior middle	662 (8.0)	562 (8.1)	100 (7.2)	
Senior middle	104 (1.3)	93 (1.3)	11 (0.8)	
College and above	14 (0.2)	12 (0.2)	2 (0.1)	
**Marital status** (N, %)				< 0.01
Single	122 (1.5)	98 (1.4)	24 (1.7)	
Married	6538 (79.1)	5515 (80.1)	1023 (74.0)	
Divorced/widowed	1609 (19.5)	1273 (18.5)	336 (24.3)	
**Family income** (N, %)				< 0.01
≤ 10,000 CNY/year	2619 (31.6)	2161 (31.3)	458 (33.1)	
10,001–20,000 CNY/year	1637 (19.8)	1362 (19.7)	275 (19.9)	
20,001–50,000 CNY/year	2125 (25.7)	1718 (24.9)	407 (29.5)	
50,001–100,000 CNY/year	1068 (12.9)	922 (13.4)	146 (10.6)	
> 100,000 CNY/year	830 (10.0)	734 (10.6)	96 (6.9)	
**BMI** (N, %)				0.17
< 18.5 kg/m^2^	420 (5.1)	339 (4.9)	81 (5.9)	
18.5 ~ < 24 kg/m^2^	4537 (55.0)	3802 (55.4)	735 (53.1)	
≥ 24 kg/m^2^	3289 (39.9)	2721 (39.7)	568 (41.0)	
**Hypertension** (Presence, N, %)	3594 (43.4)	2894 (41.9)	700 (50.5)	< 0.01
**Diabetes** (Presence, N, %)	707 (8.5)	538 (7.8)	169 (12.2)	< 0.01
**CHD** (Presence, N, %)	245 (3.0)	191 (2.8)	54 (3.9)	0.02
**Smoking** (N, %)				< 0.01
Never	5668 (68.4)	4649 (67.3)	1019 (73.6)	
Past	1793 (21.6)	1551 (22.5)	242 (17.5)	
Current	828 (10.0)	704 (10.2)	124 (9.0)	
**Alcohol drinking** (N, %)				< 0.01
Never	2300 (27.7)	1984 (28.7)	316 (22.8)	
Past	648 (7.8)	520 (7.5)	128 (9.2)	
Current	5341 (64.4)	4400 (63.7)	941 (67.9)	
**Exercise** (yes, N, %)	1642 (19.8)	1314 (19.0)	328 (23.7)	< 0.01
**Tea drinking** (yes, N, %)	2130 (25.7)	1801 (26.1)	329 (23.8)	0.07
**Vegetable intake** (≥7 days/week, N, %)	6671 (80.5)	5587 (80.9)	1084 (78.3)	0.02
**Fruit intake** (≥3 days/week, N, %)	3257 (39.3)	2803 (40.6)	454 (32.8)	< 0.01
**Red meat intake** (≥3 days/week, N, %)	4147 (50.0)	3528 (51.1)	619 (44.7)	< 0.01
**Fish intake** (≥3 days/week, N, %)	3214 (38.8)	2497 (36.2)	717 (51.8)	< 0.01

Data for the following are missing: 20 for marital status, 10 for family income, 43 for BMI, and 4 for fish intake

*Abbreviations*: *BMI* Body mass index, *CHD* Coronary heart disease, *CNY* Chinese Yuan, *SD* Standard deviation

Table 2 demonstrates the results of the multivariable analyses for the association between egg consumption and the risk of depressive symptoms. In a crude analysis (model 1), participants consuming < 3 eggs/week and ≥ 3 eggs/week showed significantly lower risks of depressive symptoms contrary to non-consumers or less-than-weekly consumers. In the models adjusting for sociodemographic characteristics and other covariates, the protective effect was also significant. The fully adjusted RRs were 0.70 (95% CI: 0.62–0.80) and 0.62 (95%CI 0.54–0.71) for participants consuming < 3 eggs/week and ≥ 3 eggs/week, respectively. Meanwhile, a linear trend was identified in the analyses (P for trend < 0.01). In addition, each egg increment per week was associated with a 4% lower risk of depressive symptoms (RR = 0.96, 95%CI 0.93–0.99).

**Table 2:** Longitudinal association between egg consumption and risk of depressive symptoms

Egg consumption	Total	Cases	Model 1^***a***^		Model 2^***b***^		Model 3^***c***^		Model 4^***d***^	
			RR (95% CI)	***P*** value	RR (95% CI)	***P*** value	RR (95% CI)	***P*** value	RR (95% CI)	***P*** value
None or not weekly	2826	629	1.00 (Ref)	–	1.00 (Ref)	–	1.00 (Ref)	–	1.00 (Ref)	–
< 3 eggs/week	2565	393	0.62 (0.55–0.71)	< 0.01	0.65 (0.57–0.73)	< 0.01	0.66 (0.58–0.74)	< 0.01	0.70 (0.62–0.80)	< 0.01
≥3 eggs/week	2898	363	0.50 (0.44–0.57)	< 0.01	0.53 (0.46–0.60)	< 0.01	0.55 (0.48–0.63)	< 0.01	0.62 (0.54–0.71)	< 0.01
P for trend			< 0.01		< 0.01		< 0.01		< 0.01	
RR for 1 egg/week			0.93 (0.90–0.96)	< 0.01	0.94 (0.91–0.97)	< 0.01	0.94 (0.92–0.97)	< 0.01	0.96 (0.93–0.99)	< 0.01

*Abbreviations*: *RR* Relative risk, *CI* Confidence interval, *Ref* Reference

^a^No variable was adjusted in model 1

^b^Adjusted for age, gender, race, education level, marital status, and family income

^c^Adjusted for the same covariates in model 2, plus body mass index, hypertension, diabetes, coronary heart disease, smoking, alcohol drinking, exercise, and tea drinking

^d^Adjusted for the same covariates in model 3, plus vegetable intake, fruit intake, red meat intake, and fish intake

To determine the potential effect modification caused by the frequency of other food intakes, we conducted stratified analyses and the results are shown in Table 3. For the subgroup with fish intake less than 3 days per week, < 3 eggs/week was not associated with the risk of depressive symptoms (P for interaction < 0.01). Moreover, there was no obvious difference across the subgroups for vegetables, fruits, and red meat.

**Table 3:** Adjusted RR (95% CI) for depressive symptoms with egg consumption, stratified by frequency of other food intakes

Subgroups	Total	Cases	Egg consumption		
			None or not weekly	< 3 eggs/week	≥3 eggs/week
**Vegetable intake**					
< 7 days/week	1617	301	1.00 (Ref)	0.59 (0.44–0.78)	0.73 (0.52–1.00)
≥ 7 days/week	6667	1081	1.00 (Ref)	0.77 (0.67–0.88)	0.62 (0.53–0.71)
P for interaction	0.10				
**Fruit intake**					
< 3 days/week	5027	928	1.00 (Ref)	0.71 (0.61–0.82)	0.66 (0.56–0.78)
≥ 3 days/week	3257	454	1.00 (Ref)	0.68 (0.54–0.86)	0.55 (0.45–0.69)
P for interaction	0.16				
**Red meat intake**					
< 3 days/week	4141	766	1.00 (Ref)	0.74 (0.63–0.86)	0.55 (0.45–0.66)
≥ 3 days/week	4143	616	1.00 (Ref)	0.63 (0.51–0.77)	0.66 (0.55–0.79)
P for interaction	0.16				
**Fish intake**					
< 3 days/week	5070	665	1.00 (Ref)	0.92 (0.77–1.10)	0.82 (0.68–0.99)
≥ 3 days/week	3214	717	1.00 (Ref)	0.60 (0.51–0.72)	0.54 (0.45–0.66)
P for interaction	< 0.01				

*Abbreviations*: *RR* Relative risk, *CI* Confidence interval, *Ref* Reference

Adjusted for all same covariates included in model 4 (see Table 2), except the variable for stratification

The sensitivity analyses, restricting the participants to those with at least two waves of follow-up data, including the participants with depressive symptoms at baseline, or using the PHQ-9 score ≥ 9 to define depressive symptoms, showed consistent results to the main analyses. (Table 4).

**Table 4:** Sensitivity analysis for the association between egg consumption and risk of depressive symptoms in a subset of participants completing at least two waves of the follow-up survey

Egg consumption	Total	Cases	Model 1		Model 2		Model 3		Model 4	
			RR (95% CI)	***P*** value	RR (95% CI)	***P*** value	RR (95% CI)	***P*** value	RR (95% CI)	***P*** value
**Sensitivity analysis 1: in participants attending the follow-up survey at least twice**										
None or not weekly	2520	600	1.00 (Ref)	–	1.00 (Ref)	–	1.00 (Ref)	–	1.00 (Ref)	–
< 3 eggs/week	2224	360	0.60 (0.53–0.68)	< 0.01	0.63 (0.55–0.71)	< 0.01	0.64 (0.56–0.72)	< 0.01	0.69 (0.61–0.78)	< 0.01
≥ 3 eggs/week	2507	346	0.51 (0.44–0.58)	< 0.01	0.54 (0.47–0.61)	< 0.01	0.56 (0.49–0.63)	< 0.01	0.63 (0.55–0.72)	< 0.01
P for trend			< 0.01		< 0.01		< 0.01		< 0.01	
RR for 1 egg/week			0.93 (0.90–0.96)	< 0.01	0.94 (0.91–0.97)	< 0.01	0.95 (0.92–0.98)	< 0.01	0.96 (0.94–0.99)	0.01
**Sensitivity analysis 2: included the participants with depressive symptoms at baseline in GEE models**										
None or not weekly	3211	918	1.00 (Ref)	–	1.00 (Ref)	–	1.00 (Ref)	–	1.00 (Ref)	–
< 3 eggs/week	2993	712	0.79 (0.71–0.86)	< 0.01	0.82 (0.75–0.90)	< 0.01	0.83 (0.75–0.91)	< 0.01	0.85 (0.77–0.93)	< 0.01
≥ 3 eggs/week	3219	568	0.57 (0.52–0.64)	< 0.01	0.61 (0.55–0.68)	< 0.01	0.64 (0.57–0.71)	< 0.01	0.71 (0.64–0.79)	< 0.01
P for trend			< 0.01		< 0.01		< 0.01		< 0.01	
RR for 1 egg/week			0.94 (0.92–0.96)	< 0.01	0.95 (0.93–0.97)	< 0.01	0.96 (0.94–0.98)	< 0.01	0.97 (0.95–0.99)	< 0.01
**Sensitivity analysis 3: using the PHQ-9 score ≥ 9 to define depressive symptoms**										
None or not weekly	3021	286	1.00 (Ref)	–	1.00 (Ref)	–	1.00 (Ref)	–	1.00 (Ref)	–
< 3 eggs/week	2765	174	0.65 (0.54–0.79)	< 0.01	0.69 (0.57–0.84)	< 0.01	0.71 (0.59–0.86)	< 0.01	0.76 (0.63–0.92)	< 0.01
≥ 3 eggs/week	3016	160	0.53 (0.43–0.64)	< 0.01	0.58 (0.47–0.70)	< 0.01	0.61 (0.50–0.74)	< 0.01	0.71 (0.58–0.86)	< 0.01
P for trend			< 0.01		< 0.01		< 0.01		< 0.01	
RR for 1 egg/week			0.89 (0.85–0.94)	< 0.01	0.91 (0.87–0.95)	< 0.01	0.92 (0.88–0.96)	< 0.01	0.95 (0.90–0.99)	0.02

*Abbreviations*: *RR* Relative risk, *CI* Confidence interval, *Ref* Reference

Variables in model 1, model 2, model 3, and model 4 were the same as those in Table 2

## Discussion

In this cohort study, we found that egg consumption was prospectively related to a reduced risk of depressive symptoms among the Chinese elderly. Notably, each egg increment per week was associated with a 4% lower risk of depressive symptoms. Sensitivity analyses indicated that the results were robust. For the subgroup with fish intake less than 3 days per week, < 3 eggs/week was not associated with the risk of depressive symptoms.

Our current findings are partially consistent with the previous epidemiological studies. A cross-sectional study [11], investigating a total of 9965 Iranian adults (20–69 years old), suggested that eating eggs more than once per week had protective effects on depressive symptoms. Unfortunately, the study only included the frequency of egg intake, while the quantity was lack of consideration. Meanwhile, the results from the SEPAHAN project [12] involving 3172 healthcare workers concluded that egg consumption was not associated with depressive symptoms. However, the study sample might not be representative of the general population, which limited the generalization of results. In a sample of 27,162 Canadian adults aged 45–85 years old, intake of omega-3 eggs was found associated with depressive symptoms in the subgroup of males [13]. Nevertheless, results about all egg dishes except omega-3 eggs were not reported in this study. Due to the cross-sectional nature of all previous studies, causal relationships between egg intake and depressive symptoms cannot be inferred. They are unable to conclude whether egg consumption alters the risk of depressive symptoms or presence of depressive symptoms leads to a behavioral change in egg consumption. In contrast with previous studies, our study was based on a longitudinal study design and had a relatively long duration of follow-up, which made our results more reliable. Moreover, our study not only quantitatively assessed egg consumption, but also conducted stratified analyses to test whether observed associations varied by other food intakes, which was not reported in the previous studies.

The mechanism responsible for the association is unknown. The risk of depression might be influenced by egg consumption through its biologically active components. Eggs contain the highest nutritional quality protein, providing all the essential amino acids in amounts that best match human requirements. Tryptophan, an essential amino acid in eggs, can exert an antidepressant effect by influencing the production of serotonin (5-HT) [21], a neurotransmitter that can improve mood and feelings of relaxation [22]. Egg yolks are one of a handful of foods that naturally contain vitamin D and vitamin B. Vitamin D appears to modulate several neurotransmitter systems, including those involved in the pathogenesis of depression [23]. Vitamin B_2_ and Vitamin B_12_ play a role in one-carbon metabolism, which in turn exerts an important function in mood regulation [24, 25]. Magnesium (Mg) is naturally rich in eggs. It is indicated that low magnesium status is associated with increased inflammatory and oxidative stress [26], which are events associated with depression. Eggs are an edible source of Omega-3 fatty acids. The antidepressant action of Omega-3 fatty acids includes reduction of inflammation, effect on 5-HT and dopamine, impact upon protein kinase C (PKC), regulation of gene expression, etcetera [27]. In addition, egg consumption has been reported with beneficial effects on cognitive function [15], and dysfunctional cognition is one of the risk factors for depression [28].

The association of lower egg consumption with depressive symptoms was indicated as insignificant among participants with lower frequency of fish intake, and the reason remained unclear. Fish own a high content of polyunsaturated fatty acids (PUFAs) and other bioactive substances such as high-quality protein, vitamins, and minerals, that have protective effects against depression [29]. A meta-analysis of observational studies demonstrated that fish intake was associated with a reduced risk of depression [30]. It can be hypothesized that eating fish has an important role, and the protective effect of egg consumption on the risk of depressive symptoms may be weakened when the fish intake is insufficient.

There are several methodological issues and limitations in this study. First, the quantity of egg intake was only inquired at baseline. Consequently, it did not allow us to assess the changes in egg consumption and its effect on the risk of depressive symptoms. Second, recall bias might exist considering the self-report of egg consumption. We could not exclude the potential misclassification, which is probably non-differential, causing the associations to attenuate toward the null. Third, no biomarker of egg intake was taken into consideration, so the observed association cannot be ascribed to any specific component among eggs in our study. Fourth, no information on antidepressant treatment was collected, which might affect our results. Last, as this study focusing the elderly with mild to severe depressive symptoms, a PHQ-9 cut-off score ≥ 5 was used, which might not have the best screening properties for detecting depression. However, we conducted a sensitivity analysis using a cut-off score ≥ 9 that is generally accepted for screening depression in China, and the results were consistent with the main analyses.

Despite the limitations, this study possesses several strengths. (1) To our best knowledge, this study was the first cohort study to examine the prospective association of egg consumption with depressive symptoms. Based on the longitudinal study design and relatively long follow-up duration, this study was able to evaluate the temporal relationship between exposure and outcome. (2) The large sample size made our results relatively robust, which might be potentially associated with a broader population of Chinese elderly. (3) Our analyses adjusted a wide range of potential confounders, involving sociodemographic characteristics, health conditions, behavioral lifestyle, and dietary habits. (4) The missing number of key variables (depressive symptoms and egg consumption) was very low because missing data and logical errors were fed back to the initial interviewers who would try to complete the dataset by reinvestigating the participants.

In conclusion, this community-based cohort study indicates that egg consumption is prospectively associated with a lower risk of depressive symptoms in the Chinese elderly. A balanced diet, including appropriate egg intake, can go a long way toward supporting better mental health practices for the elderly. The finding needs to be verified in other prospective studies before recommendations for lifestyle interventions to prevent depression can be made. Meanwhile, clinical studies are needed to test the nutrition components of eggs that benefit mental health.

## Supplementary Information

**Additional file 1: Table S1.** Baseline characteristics of 8289 included participants according to egg consumption.

## Data Availability

The datasets analyzed during the current study are not publicly available due to data regulations and ethical reasons but are available from the corresponding author upon reasonable request.

## Abbreviations

BMI: Body mass index
CHD: Coronary heart disease
CNY: Chinese yuan
SD: Standard deviation
RR: Relative risk
CI: Confidence interval
MMSE: Mini-mental state examination
PHQ-9: Patient health questionnaire-9 scale
GEE: Generalized estimating equations

## References

[1] Smith K (2014). Mental health: a world of depression. Nature.

[2] World Health Organization (2022). Depression. Fact sheet N°369 edition.

[3] Mathers CD, Loncar D (2006). Projections of global mortality and burden of disease from 2002 to 2030. PLoS Med.

[4] Blazer DG (2003). Depression in late life: review and commentary. J Gerontol A Biol Sci Med Sci.

[5] Manosso LM, Moretti M, Rodrigues AL (2013). Nutritional strategies for dealing with depression. Food Funct.

[6] Kovacs-Nolan J, Phillips M, Mine Y (2005). Advances in the value of eggs and egg components for human health. J Agric Food Chem.

[7] Pooyan S, Rahimi MH, Mollahosseini M, Khorrami-Nezhad L, Nasir Y, Maghbooli Z, Mirzaei K (2018). A high-protein/low-fat diet may interact with vitamin D-binding protein gene variants to moderate the risk of depression in apparently healthy adults. Lifestyle Genom.

[8] Gougeon L, Payette H, Morais JA, Gaudreau P, Shatenstein B, Gray-Donald K (2016). Intakes of folate, vitamin B6 and B12 and risk of depression in community-dwelling older adults: the Quebec longitudinal study on nutrition and aging. Eur J Clin Nutr.

[9] Trebaticka J, Dukat A, Durackova Z, Muchova J (2017). Cardiovascular diseases, depression disorders and potential effects of omega-3 fatty acids. Physiol Res.

[10] Nagasawa M, Otsuka T, Ogino Y, Yoshida J, Tomonaga S, Yasuo S, Furuse M (2014). Orally administered whole egg demonstrates antidepressant-like effects in the forced swimming test on rats. Acta Neuropsychiatr.

[11] Sangsefidi ZS, Mirzaei M, Hosseinzadeh M (2020). The relation between dietary intakes and psychological disorders in Iranian adults: a population-based study. BMC Psychiatry.

[12] Shafiei F, Keshteli AH, Pouraram H, Afshar H, Salari-Moghaddam A, Esmaillzadeh A, Adibi P (2019). Egg consumption and prevalence of psychological disorders in adults. Eur J Nutr.

[13] Davison KM, Lung Y, Lin SL, Tong H, Kobayashi KM, Fuller-Thomson E (2019). Depression in middle and older adulthood: the role of immigration, nutrition, and other determinants of health in the Canadian longitudinal study on aging. BMC Psychiatry.

[14] Miranda JM, Anton X, Redondo-Valbuena C, Roca-Saavedra P, Rodriguez JA, Lamas A, Franco CM, Cepeda A (2015). Egg and egg-derived foods: effects on human health and use as functional foods. Nutrients.

[15] Li FD, Tong ZD, Chang Y, Li KF, Gu X, Zhang T, Lin JF (2022). Eggs consumption in relation to lower risk of cognitive impairment in elderly: findings from a 6-year cohort study. J Nutr Health Aging.

[16] Katzman R, Zhang MY (1988). Ouang-Ya-Qu, Wang ZY, Liu WT, Yu E, Wong SC, Salmon DP, Grant I: a Chinese version of the Mini-mental state examination; impact of illiteracy in a Shanghai dementia survey. J Clin Epidemiol.

[17] Kroenke K, Spitzer RL, Williams JB (2001). The PHQ-9: validity of a brief depression severity measure. J Gen Intern Med.

[18] Chen S, Chiu H, Xu B, Ma Y, Jin T, Wu M, Conwell Y (2010). Reliability and validity of the PHQ-9 for screening late-life depression in Chinese primary care. Int J Geriatr Psychiatry.

[19] Marschner IC, Gillett AC (2012). Relative risk regression: reliable and flexible methods for log-binomial models. Biostatistics.

[20] Deddens J, Petersen M, Lei X (2003). Estimation of prevalence ratios when PROC GENMOD does not converge. Proceedings of the 28th annual SAS users group international conference.

[21] Le Floc'H N, Otten W, Merlot E (2011). Tryptophan metabolism, from nutrition to potential therapeutic applications. Amino Acids.

[22] Berton O, Nestler EJ (2006). New approaches to antidepressant drug discovery: beyond monoamines. Nat Rev Neurosci.

[23] Eyles DW, Burne TH, McGrath JJ (2013). Vitamin D, effects on brain development, adult brain function and the links between low levels of vitamin D and neuropsychiatric disease. Front Neuroendocrinol.

[24] McNulty H, McKinley MC, Wilson B, McPartlin J, Strain JJ, Weir DG, Scott JM (2002). Impaired functioning of thermolabile methylenetetrahydrofolate reductase is dependent on riboflavin status: implications for riboflavin requirements. Am J Clin Nutr.

[25] Sarris J, Schoendorfer N, Kavanagh DJ (2009). Major depressive disorder and nutritional medicine: a review of monotherapies and adjuvant treatments. Nutr Rev.

[26] Nielsen FH (2010). Magnesium, inflammation, and obesity in chronic disease. Nutr Rev.

[27] Freeman MP (2006). Omega-3 fatty acids and perinatal depression: a review of the literature and recommendations for future research. Prostaglandins Leukot Essent Fatty Acids.

[28] Hammen C (2018). Risk factors for depression: an autobiographical review. Annu Rev Clin Psychol.

[29] Wu D, Feng L, Gao Q, Li JL, Rajendran KS, Wong JC, Kua EH, Ng TP (2016). Association between fish intake and depressive symptoms among community-living older Chinese adults in Singapore: a cross-sectional study. J Nutr Health Aging.

[30] Li F, Liu X, Zhang D (2016). Fish consumption and risk of depression: a meta-analysis. J Epidemiol Community Health.

