# Supplementary material for: Egg consumption reduces the risk of depressive symptoms in the elderly: findings from a 6-year cohort study
Source: BMC Psychiatry. 2023 Jan 17;23:44. doi: 10.1186/s12888-023-04540-2 (PMC9843850; doi:10.1186/s12888-023-04540-2)
Supplement: Supplementary file 1 — Additional file 1: Table S1. Baseline characteristics of 8289 included participants according to egg consumption. [file 12888_2023_4540_MOESM1_ESM.docx]

**Table S1.** Baseline characteristics of 8289 included participants according to egg consumption.

| **Variables** | **Total**  **(N=8289)** | **Egg consumption** | | | **P value** |
| --- | --- | --- | --- | --- | --- |
|  |  | **None or not weekly**  **(N=2826)** | **<3 eggs/week**  **(N=2565)** | **≥3 eggs/week**  **(N=2898)** |  |
| **Age** (years, Mean±SD) | 68.6±7.0 | 69.1±7.1 | 68.3±6.8 | 68.3±6.9 | <0.01 |
| **Gender** (N, %) |  |  |  |  | <0.01 |
| Male | 4174 (50.4) | 1250 (44.2) | 1317 (51.3) | 1607 (55.5) |  |
| Female | 4115 (49.6) | 1576 (55.8) | 1248 (48.7) | 1291 (44.5) |  |
| **Race** (N, %) |  |  |  |  | <0.01 |
| Han ethnicity | 8060 (97.2) | 2774 (98.2) | 2491 (97.1) | 2795 (96.4) |  |
| Minority | 229 (2.8) | 52 (1.8) | 74 (2.9) | 103 (3.6) |  |
| **Education level** (N, %) |  |  |  |  | <0.01 |
| Lower than primary | 3792 (45.8) | 1501 (53.1) | 1025 (40.0) | 1266 (43.7) |  |
| Primary | 3714 (44.8) | 1145 (40.5) | 1279 (49.9) | 1290 (44.5) |  |
| Junior middle | 662 (8.0) | 154 (5.4) | 229 (8.9) | 279 (9.6) |  |
| Senior middle | 104 (1.3) | 21 (0.7) | 27 (1.1) | 56 (1.9) |  |
| College and above | 14 (0.2) | 5 (0.2) | 4 (0.2) | 5 (0.2) |  |
| **Marital status** (N, %) |  |  |  |  | <0.01 |
| Single | 122 (1.5) | 34 (1.2) | 40 (1.6) | 48 (1.7) |  |
| Married | 6538 (79.1) | 2096 (74.3) | 2070 (80.9) | 2372 (82.1) |  |
| Divorced/widowed | 1609 (19.5) | 692 (24.5) | 448 (17.5) | 469 (16.2) |  |
| **Family income** (N, %) |  |  |  |  | <0.01 |
| ≤10,000 CNY/year | 2619 (31.6) | 926 (32.8) | 715 (27.9) | 978 (33.8) |  |
| 10,001–20,000 CNY/year | 1637 (19.8) | 557 (19.7) | 518 (20.2) | 562 (19.4) |  |
| 20,001–50,000 CNY/year | 2125 (25.7) | 732 (25.9) | 758 (29.6) | 635 (21.9) |  |
| 50,001–100,000 CNY/year | 1068 (12.9) | 251 (8.9) | 363 (14.2) | 454 (15.7) |  |
| >100,000 CNY/year | 830 (10.0) | 356 (12.6) | 209 (8.2) | 265 (9.2) |  |
| **BMI** (N, %) |  |  |  |  | <0.01 |
| <18.5 kg/m^2^ | 420 (5.1) | 139 (4.9) | 119 (4.7) | 162 (5.6) |  |
| 18.5～<24 kg/m^2^ | 4537 (55.0) | 1485 (52.7) | 1401 (54.8) | 1651 (57.5) |  |
| ≥24 kg/m^2^ | 3289 (39.9) | 1194 (42.4) | 1035 (40.5) | 1060 (36.9) |  |
| **Hypertension** (Presence, N, %) | 3594 (43.4) | 1334 (47.2) | 1145 (44.6) | 1115 (38.5) | <0.01 |
| **Diabetes** (Presence, N, %) | 707 (8.5) | 282 (10.0) | 215 (8.4) | 210 (7.2) | <0.01 |
| **CHD** (Presence, N, %) | 245 (3.0) | 86 (3.0) | 79 (3.1) | 80 (2.8) | 0.74 |
| **Smoking** (N, %) |  |  |  |  | <0.01 |
| Never | 5668 (68.4) | 2086 (73.8) | 1723 (67.2) | 1859 (64.1) |  |
| Past | 1793 (21.6) | 486 (17.2) | 563 (21.9) | 744 (25.7) |  |
| Current | 828 (10.0) | 254 (9.0) | 279 (10.9) | 295 (10.2) |  |
| **Alcohol drinking** (N, %) |  |  |  |  | <0.01 |
| Never | 2300 (27.7) | 639 (22.6) | 766 (29.9) | 895 (30.9) |  |
| Past | 648 (7.8) | 215 (7.6) | 199 (7.8) | 234 (8.1) |  |
| Current | 5341 (64.4) | 1972 (69.8) | 1600 (62.4) | 1769 (61.0) |  |
| **Exercise** (yes, N, %) | 1642 (19.8) | 585 (20.7) | 595 (23.2) | 462 (15.9) | <0.01 |
| **Tea drinking** (yes, N, %) | 2130 (25.7) | 520 (18.4) | 667 (26.0) | 943 (32.5) | <0.01 |
| **Vegetable intake** (≥7 days/week, N, %) | 6671 (80.5) | 2219 (78.5) | 1981 (77.2) | 2471 (85.3) | <0.01 |
| **Fruit intake** (≥3 days/week, N, %) | 3257 (39.3) | 1008 (35.7) | 759 (29.6) | 1490 (51.4) | <0.01 |
| **Red meat intake** (≥3 days/week, N, %) | 4147 (50.0) | 1179 (41.7) | 1160 (45.2) | 1808 (62.4) | <0.01 |
| **Fish intake** (≥3 days/week, N, %) | 3214 (38.8) | 1287 (45.6) | 823 (32.1) | 1104 (38.1) | <0.01 |

Data for the following are missing: 20 for marital status, 10 for family income, 43 for BMI, 1 for fruit intake, and 4 for fish intake.

Abbreviations: BMI, body mass index; CHD, coronary heart disease; CNY, Chinese Yuan; d/week, days per week; SD, standard deviation.
